# Recommendations for definition and determination of carry-over effects

**DOI:** 10.1155/S1463924688000380

**Published:** 1988

**Authors:** R. Haeckel

**Affiliations:** Zentralkrankenhaus Inst. für Laboratoriumsmedzin Bremen 1 D2800 Germany


Journal of Automatic Chemistry Vol. 10, No. 4 (October–December 1988), pp. 181–183

### Recommendations for definition and determination of carry-over effects

**R. Haeckel**
*Zentralkrankenhaus, Inst. für Laboratoriumsmedzin, D2800 Bremen 1, FR Germany*

*The following concepts have been elaborated by the Commission on Automation and New Technologies of the International Union of Pure and Applied Chemistry [1].*

### Definitions

The term 'carry-over' is commonly used to describe a process by which materials are carried into a reaction mixture to which they do not belong. These materials can be either parts of a specimen or reagents, including diluent or wash solution. In such cases carry-over means transfer of material (specimen or reagents) from one container or from one reaction mixture to another; it can be either unidirectional or bidirectional in a series of specimens or assays.

### Classification

Carry-over can be classified according to either the material which is carried over, or to the site where the carry-over occurs, or to its dependency on the sample (see table 1).

*Table 1. Classification of carry-over effects.*

1. According to the site where it occurs: carry-over in specimen cup, sample probe, reagent probe, reaction system, signal detection system, wash station.
2. According to the material which is carried over: carry-over of specimen, diluent, reagent, reaction mixture, wash solution.
3. According to the sample dependency.
   3.1. Specimen-dependent carry-over.
   3.2. Specimen-independent carry-over.

### Current concepts for the determination of carry-over

The current concept of carry-over implies the transfer of a part of one reaction mixture to another. It is measured in terms of volume, mass or concentration fraction, either determined independently from the analytical procedure by using dye solutions or radioactive isotopes or by one of the analytical procedures applied.

In analytical chemistry, the error produced by carry-over within a particular procedure is of interest. It is dependent on the method, including all reagents applied. Therefore, carry-over effects, rather than carry-over itself, need to be determined. For this purpose, a sequence of specimens, usually with high ($a$) and low ($b$) concentrations of the analyte to be measured are processed: for example $a_1$, $a_2$, $b_1$, $b_2$, $b_3$ (see figure 1).

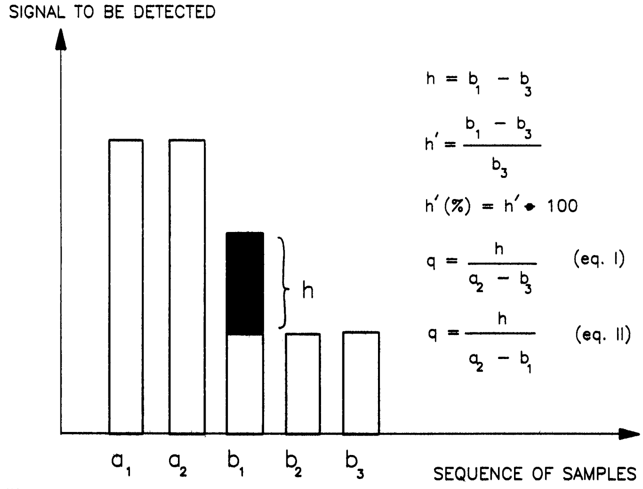

*Figure 1.*

The carry-over effect ($q$) can be calculated using either equation 1 [2]

$$q = \frac{b_1 - b_3}{a_2 - b_3} \quad (1)$$

or equation 2 [3]

$$q = \frac{b_1 - b_3}{a_2 - b_1} \quad (2)$$

$q$ may also be called the 'carry-over ratio', and is sometimes expressed as 'percentage value' ($Q = 100$ q). It implies that the influence of carry-over effects on the analytical results is inversely proportional to the concentration difference of the component to be detected by the analytical procedure in the two specimens. The last assumption is probably met in many cases [2], however, does not apply for pH/blood gas analysers and not for some selective analysers where complicated carry-over phenomena may occur from reagents to reaction mixtures.

Another disadvantage of calculating the carry-over effect ($q$) is that it does not indicate directly to what extent the resuts may be erroneously influenced. A carry-over ratio of $q = 0{\cdot}01$ ($Q = 1\%$) can lead to clinically misleading results in one case whereas a $q = 0{\cdot}1$ ($Q = 10\%$) value may be irrelevant in another case. This shall be demonstrated by two examples which are well known to clinical chemists.

In the first example $q = 0{\cdot}01$ and the ALT value following a specimen with 2000 U/l will be 40 U/l instead of 20 U/l. In the second example $q$ 10 times higher ($q = 0{\cdot}1$) and the chloride concentration following a specimen with 140

mmol/l will be 86 mmol/l instead of 80 mmol/l. In one situation the carry-over effect leads erroneously to a pathological value with clinical relevance, whereas in the other example the carry-over is clinically unimportant although carry-over is 10 times higher.

### Proposed concept for determining carry-over effects

Since the carry-over in an analytical system depends partly on the reagents, carry-over effects in a spectrometer (chosen as an example) should be determined with the reagents used in the cuvette or flow cell of a particular spectrometer rather than with dye solutions.

These effects should be expressed in units (for example mol/l or U/l) which are used to present the results and not as quantity fractions. As already mentioned, carry-over effects can be either specimen related or specimen independent.

### Specimen-dependent carry-over

Specimen-dependent carry-over either may occur with one method using a constant sample volume or in multitest analysers with sample volumes varying from method to method. Only the first case is considered; the second case is explained in reference [1].

Where the determination of a specimen with a high concentration of analyte interferes with that of a specimen with a low concentration (or vice versa), a sequence of at least two successive aliquots of a specimen with a high value (*a*), followed by at least three successive aliquots of one with a low value (*b*), should be used to determine the carry-over effect.

The concentration of (*b*) should be chosen to be close to the most relevant decision level, which in many cases is the upper limit of the reference interval (for example 20–30 U/l in the case of aminotransferase catalytic activity).

The concentration of (*a*) should represent the extreme values which may occur (for example 1000–2000 U/l for aminotransferase activity concentration or 50 mmol/l for glucose concentration). The volume in the container from which the sample is taken must be defined (usually it should be filled to two-thirds of its maximum capacity).

This experiment should be repeated 10 times [5]. For the comparison of the paired values $b_1$ ($\bar{b}_1$) and $b_3$ ($\bar{b}_3$) the Wilcoxon signed rank test is recommended. If a highly significant (for example $\alpha \leq 1\%$) difference is detected, then the mean carry-over effect (*h*) is calculated see (figure 1):

$$h = \bar{b}_1 - \bar{b}_3 \qquad (3)$$

The carry-over effect may also be expressed as a fraction of $b_3$ or as percentage of the value $b_3$.

When the carry-over effect is not highly significant, the procedure can be classified as carry-over safe in an interval between the quantity measured in specimen *b* (lower limit) and the quantity measured in specimen *a* (upper limit). After a carry-over effect has been detected, it is common practice to repeat a test when the difference between its result and the previous one suggests that a significant error has occured due to carry-over effects. Which difference can just be tolerated (that means the carry-over safe interval) is usually estimated by intuition. However, it can also be determined experimentally. For this purpose, the experiment reported above must be repeated with lower (*a*)-value(s) which are close to the upper limit of the linear range. If a significant carry-over effect cannot be detected, the upper limit of the carry-over safe interval is assumed to be identical with the upper limit of the linearity interval. If the carry-over effect is still significant, then the concentration at the upper limit of the carry-over safe interval can be estimated. It shall be assumed that

$$\bar{b}_1 - \bar{b}_3 \leq \bar{b}_3 + 2s$$

This means that the carry-over effect $h = \bar{b}_1 - \bar{b}_3$ should be smaller than twice the standard deviation obtained with this particular method. Furthermore it is assumed that the carry-over effect increases linearily with the difference of quantities measured in specimens *a* and *b*. The carry-over safe interval can then be calculated by the equation

$$a_s = \frac{2s\,(1 + q)}{q} + \bar{b}_3 \qquad (5)$$

where *s* is the standard deviation calculated from the 10 $b_3$-values determined in the previous carry-over experiment; *q* is calculated similarly to equation I from the results of the previous carry-over experiment (the *a* value is close to the upper limit of the linear range).

After the carry-over safe interval has been determined, all results close to the reference range of the particular analytical procedure should be regarded as falsely increased if they follow a result above the carry-over safe interval. This interval is not valid for results which are far below the *b*-value chosen for the calculation of the carry-over effect. In some cases the carry-over safe interval may be relatively small and too many analyses have to be repeated. Then, each result can be corrected by using a factor *q* calculated according to either equation I or II. However, such a situation should be avoided.

### Specimen-independent carry-over

Specimen-independent carry-over represents another type of carry-over which can be caused by diluent (diluent carry-over) or by reagents (reagent carry-over). Reagent probe carry-over may for example, be encountered in analysers where the same reagent probe is used to dispense reagents from two or more reagent containers, on a random basis.

The reagent probe carry-over can be determined from the following sequence using the same specimen and different methods (identified by letters):

$a_1, a_2, a_3, a_4, a_5, b, a_6, c, a_7, d, a_8, e, a_9, a_{10}, a_{11}, a_{12}, a_{13}, a_{14}.$

The reagent probe carry-over from reagents of method b is calculated according to equation (6):

$$h_b = a_6 - \bar{a} \quad (6)$$

The error in $a_6$ ($a_7$, $a_8$, $a_9$) arising from carry-over due to reagent $x$ in test $b$ (or $c$, $d$, $e$) can be ignored if the value of $a_6$ to $a_9$ is less than the mean value of $\bar{a} \pm 2s$: of the values determined in specimen $a$.

The sequence must be analysed for each method (*a–e* in the example chosen) performed on the particular multitest analyser and should be repeated at least once unless the instrument design makes some reagents interactions impossible. Marked effects have been reported for carry-over, for example between reagents for the lipase and triglyceride assays [3]. The specimen used should have a mid-range concentration of all the analytes to be measured so that both inhibition and enhancement effects can be detected [4].

### Conclusion

The concept of presenting carry-over effects as a difference of two results, or its corresponding percentage value, has two advantages:

(1) It is applicable to all conditions which are currently found in practice.

(2) It directly reports on how much a particular result deviates from its correct value which is obtained in the absence of carry-over effects.

Developments of new analytical systems should avoid any type of carry-over as far as possible. For this reason the concept realized by Technicon in the RA 1000 should be mentioned as an excellent example which should be followed by other companies and stimulate further improvements. The patented principle is the application of an immiscible liquid called Technicon random access fluid. This is a fluorocarbon and coats the internal and external surface of the sample and the reagent probe to prevent sample or reagent contact with the probe walls. A single probe is used for the aspiration and dispensing of all samples while another handles all reagents.

This principle has been further developed to the so-called capsule flow technique applied in the new Chem-1 analyser which seems to introduce a new generation of exciting analytical systems.

### References

1. Haeckel, R., Publication in preparation.
2. Haeckel, R., and Porth, J. A., *Journal of Clinical Chemistry and Biochemistry*, **10** (1972), 91.
3. Haeckel, R., *Journal of Clinical Chemistry and Biochemistry*, **23** (1985), 225.
4. Broughton, P. M. G., Gownlock, A. H., McCormack, J. J. and Neill, D. W., *Annals of Clinical Biochemistry*, **11** (1974), 207.

---

**TECHNICON H* 1/H6000 USERS' MEETING**

The 1988 H*1/H6000 Users' Meeting was held in Oxford, UK. Users were brought up-to-date with the latest modifications for the H*1 – these included an IDEE reader, automatic sampler and additional data storage. David Powell from J. S. Pathology Services then related two years' comparison of the two H*1s, the H6000 and two Coulters on that site. The 'Dumping Provocation Test' was the topic for Neil Porter's presentation. He has used the H6000 at the Royal Hallamshire Hospital to monitor the sudden decrease in PCV that can occur in some patients following gastric surgery. Dr Martin Rowan of the Western Infirmary, Glasgow, spoke about the validity of the MCV and MCHC and Keith Morris from Coventry gave results of a study relating to the use of the H*1 in iron deficiency and spherocytosis. Dr John Van de Pette gave the first of three presentations from Frimley Park Hospital where an H*1 Junior is installed. He referred to the use of the H*1 red cell data to monitor reticulocyte response in megalobalstic anaemia. His colleagues, James Newhouse and Tony Bateman, then gave papers on the laboratory's assessment of the H*1 Junior and PCV measurements in iron deficient red cells. The day finished with presentations on monitoring the progress of AIDS patients and measuring haemoglobin in the presence of gross leucocytosis.

The first paper of the second day was presented by Mike Watts from The Middlesex Hospital who had spent hours doing manual differentials on leucopenic samples and compared them with the results obtained from the H*1. Following on from this, Dr John Richards from University College Hospital spoke of the blast flag response after bone marrow transplantation. Sheila Worthington from George Eliot Hospital described tracking down the members of a family in Nuneaton who exhibited beta thalassaemia trait. The final H*1 User to speak was Tom Cavanagh from Glasgow who presented a comparison of the three part and five part differentials.

*Further details from Technicon.*

